# Assessment of growth performance and meat quality of finishing pigs raised on the low plane of nutrition

**DOI:** 10.1186/s40781-015-0070-4

**Published:** 2015-10-05

**Authors:** Jung Seok Choi, Sang-Keun Jin, C. Young Lee

**Affiliations:** Swine Science and Technology Center, Gyeongnam National University of Science, and Technology, Jinju, 52726 South Korea; The Regional Animal Industry Center, Gyeongnam National University of Science and Technology, Jinju, 52726 South Korea

**Keywords:** Finishing pig, Plane of nutrition, Growth, Meat quality, Sensory evaluation

## Abstract

This study was performed to investigate the effects of the low plane of nutrition on growth and meat quality of finishing pigs. A total of 136 crossbred barrows and gilts weighing approximately 55 kg were allotted to 8 pens, with 17 animals housed per pen, in a 2 (sex) × 2 (nutrition) factorial arrangement of treatments. The animals allotted to a medium plane of nutrition (MPN) received a finisher phase 1 (P1) diet containing 3.47 Mcal DE/kg and 0.92 % lysine and a P2 diet containing 3.40 Mcal DE/kg and 0.78 % lysine for 35 d and 36/43 d, respectively; the animals allotted to the low plane of nutrition (LPN) received only a P2 diet containing 3.00 Mcal DE/kg and 0.68 % lysine 7 d longer than MPN. The animals were slaughtered following the feeding trial, after which the loin, ham, Boston butt, and belly were taken from a total of 24 animals, with the average live weight being 120 kg, and their physicochemical and sensory quality traits were analyzed. Average daily gain did not differ between MPN and LPN during either P1 or P2. Average daily feed intake was greater (P < 0.05) in LPN vs. MPN during both phases whereas the opposite was true for the gain:feed ratio. Backfat thickness (BFT) was less in LPN vs. MPN (21.7 vs. 24.1 mm at 115 kg). The plane of nutrition influenced no effect on any of the physicochemical characteristics of fresh loin, ham, or Boston butt analyzed in the present study. Fresh hams from LPN exhibited superior aroma and odor scores than those from MPN; however, sensory quality traits were not influenced by the plane of nutrition in other fresh primal cuts or cooked meat. Instead, fresh primal cuts and cooked meat from gilts rendered superior physicochemical characteristics and sensory scores, respectively, than those from barrows. Results suggest that the low plane of nutrition may be useful to increase the slaughter weight of finishing pigs with a moderately high BFT by virtue of its BFT-lowering effect with or without exerting a slightly positive influence on pork quality.

## Background

The market weight or slaughter weight and the plane of nutrition are important factors in pig production influencing the profitability as well as meat quality [1–4]. The profitability of pig production increases with increasing slaughter weight because the production cost per unit weight of pork decreases with increasing size of the market pig [5, 6]. However, finishing pigs over-fatten after an optimal slaughter weight resulting in a decrease in carcass quality, for which reason the pig market weight is controlled within a certain range depending on the demands of pork consumers [2, 3]. The pig slaughter weight is determined largely by genetics and nutrition in addition to the consumers’ demands. As for genetics, the pigs for pork production have been bred to maximize the lean growth and/or to minimize fat deposition for the past several decades ([7–9]; cf. 7 vs. [8]), which has resulted in a remarkable increase in slaughter weight as exemplified by an approximately 20 kg increase during the past few decades in the USA [10]. Regarding nutrition, an increase in the plane of nutrition usually results in an increase not only in lean gain but in fat deposition [11, 12], which may also limit the slaughter weight of finishing pigs with a lower lean gain rate. Conversely, the pig slaughter weight could be increased by raising finishing pigs on a low plane of nutrition, thereby restricting the growth rate and fat deposition [13–15].

It is generally known that concentrations of favor-enriching substances in meat increase with age in the pig as well as other farm animal species [16]. It was under this rationale that we investigated the effects of the low plane of nutrition for finishing pigs on physicochemical and sensory characteristics of their meat with a hypothesis that pork quality would be improved if finishing pigs are raised on a low plane of nutrition to increase their age at a predetermined slaughter weight [13, 14, 17]. In our first study using a high lean-gain line, fresh loin and/or ham from finishing pigs fed a low-energy diet containing 3.00 Mcal DE/kg from 80 kg to 110, 125, or 138 kg of body weight exhibited increased redness and marbling score than those from the animals fed a ‘medium’-energy diet containing 3.20 Mcal DE/kg [13]. The effect of the plane of nutrition on sensory attributes of cooked meat was not consistent among the primal cuts in that study. In a second trial using a medium lean-gain line, the redness and scores of some sensory quality traits of fresh loin, Boston butt, and ham from the pigs fed a diet containing 3.06 Mcal DE/kg and 0.67 % lysine throughout the finishing period beginning from 48 kg were greater than those from the animals finished on a high plane of nutrition [17]. Production efficiency, however, was not examined in that study, nor investigated were the effects of the low plane of nutrition relative to those of a medium plane of nutrition, which is retrospectively thought to be optimal for the line of animals used in that study. Furthermore, the animals used for meat quality analyses, which were slaughtered at 125 kg, turned out to be over-fattened as indicated by their high average backfat thickness exceeding 24 mm. The present study was therefore performed to investigate the effects of the low plane of nutrition compared with those of the medium plane for the same line of finishing pigs on their production efficiencies and meat quality at 120 kg of a predicted optimum slaughter weight.

## Methods

### Feeding and slaughtering

The experimental protocol for the present study conformed to the guideline of Institutional Animal Care and Use Committee (IACUC) at Gyeongnam National University of Science and Technology. A total of 136 112- or 113-d-old barrows and gilts born to duroc-sired (Yorkshire × Landrace) dams were selected from a large number of contemporary pigs weighing approximately 55 kg, after which the animals were allotted randomly to 8 pens, with 17 animals housed per pen, in a 2 (sex) × 2 (plane of nutrition; ‘medium’ vs. ‘low’) factorial arrangement of treatments. The barrows and gilts assigned to the medium plane of nutrition (MPN) were provided with a commercial phase 1 finisher diet and subsequently a commercial phase 2 finisher diet for 35 d and 36/43 d (in the former and latter, respectively), respectively, whereas the barrows and gilts assigned to the low plane of nutrition (LPN) received a phase 2 finisher diet containing 3.00 Mcal DE/kg and 0.68 % lysine for 79 and 86 d, respectively (Table 1). The DE density and lysine:calorie ratio of the diets provided for MPN were comparable to the NRC [8] recommendations for finishing pigs with a medium-low lean gain rate. The energy density of the low-plane phase 2 finisher diet was 12 % lower than the NRC [8] recommendation whereas the lysine:calorie ratio of the diet was comparable to the NRC [8] recommendation for finishing pigs with a low lean growth rate.

**Table 1:** Composition of the experimental finisher diets (as-fed basis)

Item	Medium plane^a^		Low plane^b^
	Phase 1	Phase 2	Phase 2
DE^c^, Mcal/kg	3.47	3.40	3.00
Crude protein^d^, %	15.22	15.10	13.80
Lysine^c^, %	0.92	0.78	0.68
Crude fat^d^, %	9.56	7.59	3.65
Crude fiber^d^, %	4.44	5.09	5.63
Crude ash^d^, %	3.79	4.28	4.61

^a^Corn and wheat-soybean meal-based commercial diets whose ingredients compositions are unknown. The DE and lysine values were kindly provided by the formulator of the diets

^b^A corn and wheat (52 %)-wheat bran (30 %)-based diet formulated for the present experiment

^c^Calculated value

^d^Analyzed value

All animals, excluding those weighing less than 100 kg, were transported to a local abattoir following measurement of their final BW, slaughtered the following day after an overnight lairage, and chilled overnight at 2 ± 2 °C. The cold carcasses were graded and fabricated according to the MAFRA [18] and MFAS [19] standards, respectively, after measurement or evaluation of carcass weight, backfat thickness, and others. The backfat thickness (BFT) at a desired live weight and the live weight at a desired backfat thickness were adjusted and predicted, respectively, using the equation suggested by NSIF [20] and the slope of BFT regressed on the live weight reported by Park and Lee [4], respectively.

### Physicochemical analysis and sensory evaluation

The loin, ham, Boston butt, and belly were taken for laboratory analyses from 6 carcasses of as many animals per experimental group which had been selected within the animals weighing approximately 120 kg at the end of the feeding trial. Physicochemical characteristics including the color, pH, drip loss, cooking loss, water holding capacity, Warner-Bratzler shear force, firmness, chewiness, and chemical composition of fresh and/or cooked meat were determined as previously described [15, 17, 21].

Sensory quality traits of fresh and cooked meat were scored according to a 9-ladder whole number scale by 7 trained panelists as previously described [6, 17, 21]. Each trait was scored in such a way that a greater score indicates a better quality, vice versa, regardless of the positive or negative nature or the trait.

### Statistical analysis

All data were analyzed using the General Linear Model procedure of SAS (DAS Inst., Inc., Cary, NC, USA). The pen nested within the plane of nutrition (treatment) × sex and the animal nested within treatment × sex were the experimental units in growth performance and *postmortem* variables, respectively. The statistical model included the treatment, sex, treatment × sex interaction, pen (treatment × sex) as fixed errors in the analysis for growth variables, whereas in the average daily feed intake and gain:feed ratio, only the main effects and their interaction were included. The main effects and their interaction were tested using the pen (treatment × sex) as the error term in growth performance. In *postmortem* variables, the model included the animal (treatment × sex) instead of the pen in addition to the main effects and their interaction, as well as the panelist in sensory evaluation, with the animal (treatment × sex) used as the error term to test the significance of the main effects and their interaction. Means were separated using the PDIFF option only when the *P*-value for either of the main effects or their interaction was less than 0.05.

## Results

### Growth performance

Average daily gain (ADG) during the 35-d finisher phase 1 (P1) did not differ between MPN and LPN or between the groups of barrows (BARROW) and gilts (GILT; Table 2). Average daily feed intake (ADFI) during P1 did not differ between BARROW and GILT. However, ADFI was greater in LPN than in MPN (P < 0.05) whereas the opposite was true for the gain:feed ratio. During varying duration of the finisher phase 2 (P2) ranging from 36 to 51 d depending on the plane of nutrition and sex, ADG did not differ between the two nutritional treatments or sexes. The ADFI during P2 was greater in LPN and BARROW than in MPN and GILT, respectively, the gain:feed ratio being greater in MPN than in LPN. Overall, ADG did not differ between the treatments or sexes during the entire finisher phase; ADFI was greater in LPN and BARROW than in their respective counterparts whereas the gain:feed ratio was greater in MPN vs. LPN.

**Table 2:** Effects of the plane of nutrition on growth performance of finishing pigs

Variable	Medium plane^a^		Low plane^b^		SEM	*P*-value		
	Barrow	Gilt	Barrow	Gilt		Nutr.	Sex	N×S
Phase 1 (P1)								
Initial wt, kg	57.4	54.0	54.7	52.1	1.4	0.18	0.10	0.80
Final wt, kg	86.4	85.0	84.1	79.2	0.9	0.01	0.03	0.13
Days on feed	35	35	35	35				
ADG, kg	0.83	0.88	0.83	0.78	0.02	0.08	0.74	0.07
ADFI, kg	2.46	2.45	2.54	2.54	0.01	<0.01	0.70	0.95
Gain:feed	0.338	0.357	0.328	0.306	0.009	0.03	0.83	0.08
Phase 2 (P2)								
Final wt, kg	112.4	117.9	116.9	114.3	1.6	0.79	0.42	0.07
Days on feed	36	43	44	51				
ADG, kg	0.73	0.75	0.74	0.69	0.03	0.49	0.65	0.30
ADFI, kg	2.57	2.51	3.04	2.80	0.05	<0.01	0.04	0.17
Gain:feed	0.284	0.300	0.245	0.247	0.008	<0.01	0.33	0.47
Overall								
ADG, kg	0.77	0.81	0.78	0.72	0.02	0.06	0.49	0.03
ADFI, kg	2.52	2.48	2.82	2.69	0.03	<0.01	0.05	0.18
Gain:feed	0.308	0.327	0.278	0.268	0.005	<0.01	0.43	0.05

^a^Fed the medium-plane P1 and P2 finisher diets (Table 1) during P1 and P2, respectively

^b^Fed the low-plane P2 finisher diet (Table 1) during both P1 and P2

^a,b^Data are means of two pens, with 17 pigs per pen

The live weight and carcass weight did not differ between MPN and LPN or between BARROW and GILT (Table 3). Dressing percentage, however, was greater in MPN than in LPN. The BFT adjusted for a 115-kg live weight also was greater in MPN vs. LPN. Conversely, the predicted live weight adjusted for a 22.5-mm BFT was greater in the latter.

**Table 3:** Effects of the plane of nutrition on carcass characteristics of finishing pigs

Variable	Medium plane^a^		Low plane^b^		*P*-value		
	Barrow	Gilt	Barrow	Gilt	Nutr.	Sex	N×S
	(*n*=32)	(*n*=31)	(*n*=29)	(*n*=32)			
Live wt, kg	114.2±1.5	117.7±1.5	117.6±1.6	115.4±1.5	0.71	0.69	0.07
Carcass wt, kg	87.2±1.3	89.9±1.3	88.6±1.3	87.2±1.2	0.60	0.59	0.11
Dressing, %	76.3±0.4	76.4±0.4	75.3±0.4	75.6±0.4	0.02	0.57	0.76
BFT^c^, mm	23.6±0.6	24.8±0.6	22.4±0.7	21.6±0.6	<0.01	0.79	0.14
BFT at 115 kg^d^, mm	24.0±0.6	24.2±0.6	21.9±0.7	21.5±0.6	<0.01	0.96	0.62
Live wt at 22.5 mm BFT^e^	109.0±2.8	107.3±2.8	118.1±3.0	119.7±2.8	<0.01	0.99	0.58

^a^Fed the medium-plane phase 1 (P1) and phase 2 (P2) finisher diets (Table 1) during P1 and P2, respectively

^b^Fed the low-plane P2 finisher diet (Table 1) during both P1 and P2

^c^Average of backfat thickness measurements between the 11^th^ and 12^th^ ribs and at the last rib

^d^BFT adjusted for a 115-kg live weight: (115 – live wt) × BFT/(live wt – 13.608)

^e^Actual live wt + (22.5 – BFT)/0.22

### Physicochemical characteristics of the primal cuts

None of the live weight, carcass weight, dressing percentage, and BFT of the animals selected for laboratory analyses was different between the two groups differing in the plane of nutrition or sex (Table 4). In fresh loin, neither the CIE [22] L* (lightness) and a* (redness) values of the longissimus muscle (LM) nor the L*, b* (yellowness) and W (whiteness) values of the backfat covering LM differed between MPN and LPN or between BARROW and GILT. The pH value and water holding capacity (WHC) of LM also were not influenced by the treatment or sex. Drip loss and shear force values for LM and backfat were greater in BARROW than in GILT. In chemical composition of LM, moisture content was greater in GILT vs. BARROW only within LPN. Fat content of LM was greater in BARROW vs. GILT, which was more evident within LPN than within MPN. Protein content, however, did not differ between the treatments or sexes. In cooked LM, the shear force value was greater in BARROW than in GILT, but cooking loss, firmness, and chewiness were not influenced by the plane of nutrition or sex.

**Table 4:** Physicochemical characteristics of the loin, Boston Butt, and ham from the finishing pigs that were placed on either the medium or low plane of nutrition

Variable	Medium plane^a^		Low plane^b^		SEM	*P*-value		
	Barrow	Gilt	Barrow	Gilt		Nutr.	Sex	N×S
Whole carcass								
Live wt, kg	120.6	120.6	120.1	120.4	1.1	0.75	0.85	0.89
Carcass wt, kg	92.5	91.5	90.8	92.7	0.9	0.78	0.64	0.12
Dressing, %	76.7	75.9	75.7	76.9	0.7	0.99	0.75	0.15
BFT^d^, mm	23.9	22.5	24.7	22.2	1.5	0.88	0.23	0.75
Loin^c^								
CIE L*	52.1	51.9	53.7	50.4	1.4	0.98	0.23	0.29
CIE a*	8.21	7.51	6.98	6.45	0.56	0.06	0.29	0.88
CIE L* (fat)	81.5	82.0	82.6	82.1	0.4	0.12	0.92	0.20
CIE b* (fat)	3.10	3.15	3.15	3.42	0.27	0.29	0.90	0.94
CIE W* (fat)	72.2	72.5	72.3	71.8	0.8	0.73	0.94	0.61
pHu	5.75	5.74	5.70	5.79	0.06	0.98	0.54	0.43
Drip loss, %	5.38	2.77	6.19	1.68	0.96	0.89	<0.01	0.34
WHC^d^	67.8	65.4	66.5	66.8	0.9	0.95	0.27	0.18
W-B SF^d^, kg/cm^2^	5.42	3.30	4.59	2.93	0.44	0.19	<0.01	0.61
W-B SF (fat), kg/cm^2^	4.10	3.15	4.19	2.22	0.49	0.40	<0.01	0.31
Moisture, %	74.0	73.5	73.6	74.3	0.2	0.31	0.71	<0.01
Fat, %	3.01	2.37	3.68	2.03	0.20	0.42	<0.01	0.02
Protein, %	24.82	23.23	23.24	24.01	1.43	0.78	0.78	0.42
Cooking loss, %	36.2	36.1	36.1	36.6	0.6	0.82	0.76	0.63
W-B SF (cooked), kg/cm^2^	5.09	4.43	5.17	4.54	0.18	0.61	<0.01	0.92
Firmness (cooked), kg/cm^2^	1.51	1.37	1.48	1.42	0.10	0.90	0.31	0.69
Chewiness (cooked), kg	0.69	0.59	0.67	0.72	0.10	0.60	0.82	0.49
Ham^e^								
CIE L*	50.4	50.2	49.2	47.6	1.5	0.21	0.53	0.65
CIE a*	9.76	10.47	11.11	9.10	0.82	0.99	0.44	0.11
CIE L* (fat)	50.4	50.2	49.2	47.6	1.5	0.21	0.53	0.65
CIE b* (fat)	3.66	3.51	3.76	2.24	0.56	0.31	0.15	0.24
CIE W* (fat)	39.5	39.7	38.0	40.9	1.0	0.89	0.15	0.20
pHu	5.75	5.80	5.72	5.96	0.07	0.37	0.04	0.17
Drip loss, %	3.24	1.45	3.91	2.25	0.75	0.34	0.03	0.94
WHC	62.1	63.9	63.0	64.6	1.2	0.49	0.16	0.92
W-B SF, kg/cm^2^	4.23	2.69	3.90	3.65	0.37	0.41	0.02	0.09
W-B SF (fat), kg/cm^2^	4.83	3.81	3.86	4.69	0.49	0.93	0.84	0.07
Moisture, %	74.5	74.6	74.5	74.8	0.2	0.55	0.34	0.51
Fat, %	2.10	2.72	2.41	2.71	0.19	0.43	0.03	0.43
Protein, %	23.62	22.09	21.96	23.45	1.30	0.91	0.99	0.26
Cooking loss, %	33.3	35.0	33.6	35.2	0.7	0.75	0.03	0.95
W-B SF (cooked), kg/cm^2^	5.53	4.28	5.99	4.25	0.26	0.42	<0.01	0.36
Firmness (cooked), kg/cm^2^	1.05	1.19	1.13	1.23	0.08	0.45	0.17	0.82
Chewiness (cooked), kg	0.44	0.51	0.51	0.56	0.05	0.22	0.20	0.80
Boston butt^3^								
CIE L*	49.9	46.5	49.9	44.2	1.4	0.43	<0.01	0.42
CIE a*	15.26	13.38	14.67	12.32	0.69	0.25	<0.01	0.74
CIE L* (fat)	49.9	46.5	49.9	44.2	1.4	0.43	<0.01	0.42
CIE b (fat)	4.95	4.49	5.03	3.33	0.39	0.18	0.01	0.13
CIE W* (fat)	24.6	33.0	22.9	34.2	1.4	0.84	<0.01	0.31
pHu	6.25	6.44	6.23	6.56	0.06	0.38	<0.01	0.24
Drip loss, %	0.34	0.37	0.52	0.32	0.05	0.20	0.13	0.03
WHC	58.3	62.8	57.1	60.0	2.8	0.48	0.20	0.79
W-B SF, kg/cm^2^	3.48	2.86	3.50	2.37	0.28	0.41	<0.01	0.37
W-B SF (fat), kg/cm^2^	4.47	6.64	4.78	4.46	0.63	0.15	0.16	0.06
Cooking loss (cooked), %	35.9	35.5	37.1	33.5	0.7	0.58	<0.01	0.03
W-B SF (cooked), kg/cm^2^	5.92	4.83	5.78	5.14	0.45	0.86	0.07	0.61
Firmness (cooked), kg/cm^2^	1.26	1.24	1.17	12.17	0.11	0.45	0.90	0.95
Chewiness (cooked), kg	0.57	0.58	0.58	0.56	0.06	0.95	0.90	0.78

^a^Fed the medium-plane phase 1 (P1) and phase 2 (P2) finisher diets (Table 1) during P1 and P2, respectively

^b^Fed the low-plane P2 finisher diet (Table 1) during both P1 and P2

^a,b^Data are means of 6 animals

^c,e^Longissimus muscle and semimembranosus muscle, respectively, were used for the measurement of all variables except for those with an indication “fat” in parenthesis in which subcutaneous fat was used

^d^BFT, average of backfat thickness measurements between the 11^th^ and 12^th^ ribs and at the last rib adjusted for a 115-kg live weight; WHC, water holding capacity; W-B SF, Warner-Bratzler shear force

In fresh ham, none of the color variables of the semimembranosus muscle (SM) and subcutaneous fat measured in the present study differed between the treatments or sexes. The pH value of SM was greater in GILT vs. BARROW whereas the drip loss and shear force value for the muscle were greater in the latter. However, the shear force value for subcutaneous fat and WHC and protein content of SM did not differ between the treatments or sexes. Fat content of SM was greater in GILT than in BARROW, but the sex effect was significant only within MPN. In cooked SM, cooking loss was greater in GILT vs. BARROW, but the shear force value was greater in the latter. The firmness and chewiness, however, were not influenced by the treatment or sex.

In fresh Boston butt, the L* and a* values of LM as well as the L* and b* values of the subcutaneous fat were greater in BARROW than in GILT whereas the W value of the fat was greater in the latter. Drip loss of LM was greater in BARROW vs. GILT only within LPN. The WHC and shear force value for the subcutaneous fat were not influenced by the plane of nutrition or sex, but the shear force value for LM was greater in BARROW vs. GILT. In cooked LM, cooking loss was greater in BARROW vs. GILT, with the sex effect being significant only within LPN. However, none of the effects of the treatment, sex, and their interaction was significant in the shear force value, firmness, or chewiness. Finally, the ratio of the muscle area to fat area of the belly slice at the 11^th^ rib also was not influenced by the treatment or sex (data not shown).

### Sensory attributes

The color, aroma, and off-odor scores were not influenced by either the treatment or sex in sensory evaluation for fresh loin (Table 5). The drip score was greater in BARROW than in GILT, but marbling score was greater in the latter. Overall acceptability score for fresh loin was not different between the treatments or sexes.

**Table 5:** Effects of the plane of nutrition for finishing pigs on their sensory quality traits^a^

Variable	Medium plane^b^		Low plane^c^		SEM	*P*-value		
	Barrow	Gilt	Barrow	Gilt		Nutr.	Sex	N×S
Loin								
Color	7.05	6.88	6.79	7.29	0.17	0.68	0.34	0.07
Aroma	6.64	6.56	6.36	6.73	0.14	0.67	0.31	0.11
Off-odor	6.70	6.64	6.13	6.83	0.19	0.33	0.10	0.06
Drip	7.10	6.76	6.86	6.61	0.10	0.07	0.01	0.69
Marbling	6.51	7.05	6.53	6.87	0.19	0.67	0.03	0.63
Acceptability	7.00	6.82	6.70	7.06	0.15	0.85	0.57	0.10
Ham								
Color	6.56	6.51	6.75	7.10	0.17	0.04	0.40	0.27
Aroma	6.51	5.55	6.31	6.15	0.20	0.33	0.01	0.06
Off-odor	6.46	5.23	6.26	6.05	0.14	0.04	<0.01	<0.01
Drip	6.81	6.31	6.67	6.58	0.16	0.68	0.08	0.21
Marbling	4.27	4.42	4.55	4.69	0.17	0.13	0.42	1.00
Acceptability	6.60	6.19	6.48	6.68	0.11	0.12	0.38	0.01
Boston butt								
Color	6.77	6.85	6.73	7.19	0.18	0.42	0.16	0.29
Aroma	6.93	7.01	6.61	7.20	0.16	0.69	0.05	0.13
Off-odor	6.89	7.19	6.90	7.33	0.16	0.64	0.04	0.69
Drip	7.26	6.85	6.87	7.05	0.14	0.50	0.40	0.04
Marbling	6.82	6.71	6.79	6.71	0.22	0.94	0.69	0.94
Acceptability	7.15	6.77	6.90	7.00	0.15	0.94	0.36	0.13
Belly								
Color	7.23	7.15	7.24	7.07	0.09	0.70	0.20	0.60
Aroma	7.17	7.18	7.08	7.45	0.12	0.43	0.12	0.14
Off-odor	6.82	6.17	6.58	6.70	0.26	0.57	0.31	0.15
Drip	6.60	6.19	6.48	6.76	0.22	0.31	0.79	0.13
Acceptability	7.31	6.80	7.23	7.00	0.12	0.59	<0.01	0.27

^a^Scored by 7 panelists according to a 9-ladder whole number scale such that a greater score indicates a better quality in all traits

^b^Fed the medium-plane phase 1 (P1) and phase 2 (P2) finisher diets (Table 1) during P1 and P2, respectively

^c^Fed the low-plane P2 finisher diet (Table 1) during both P1 and P2

^b,c^Data are means of 6 animals

**Table 6:** Sensory quality traits of cooked meats from the finishing pigs that were placed on either the medium or low plane of nutrition^a^

Variable	Medium plane^b^		Low plane^c^		SEM	*P*-value		
	Barrow	Gilt	Barrow	Gilt		Nutr.	Sex	N×S
Loin								
Color	7.02	6.80	6.98	6.85	0.08	1.00	0.04	0.57
Aroma	6.81	6.73	6.76	6.83	0.07	0.67	0.93	0.28
Taste	6.42	6.46	6.46	6.49	0.10	0.71	0.71	0.90
Juiciness	5.95	6.25	6.18	6.40	0.11	0.11	0.03	0.76
Tenderness	5.65	6.50	5.98	6.42	0.14	0.39	<0.01	0.15
Acceptability	6.31	6.57	6.41	6.74	0.12	0.27	0.02	0.76
Ham								
Color	6.88	6.80	6.77	7.04	0.08	0.43	0.29	0.05
Aroma	6.63	7.15	6.73	7.04	0.07	0.86	<0.01	0.12
Taste	6.58	7.04	6.61	7.02	0.09	0.95	<0.01	0.85
Juiciness	6.20	6.92	6.55	6.99	0.15	0.18	<0.01	0.37
Tenderness	6.00	6.79	5.86	7.19	0.20	0.52	<0.01	0.19
Acceptability	6.51	7.12	6.50	7.10	0.11	0.87	<0.01	0.96

^a^Scored by 7 panelists according to a 9-ladder whole number scale such that a greater score indicates a better quality in all traits

^b^Fed the medium-plane phase 1 (P1) and phase 2 (P2) finisher diets (Table 1) during P1 and P2, respectively

^c^Fed the low-plane P2 finisher diet (Table 1) during both P1 and P2

^b,c^Data are means of 6 animals

In fresh ham, the color and off-odor scores were greater in LPN vs. MPN, but none of the other sensory quality traits scored in the present study was influenced by the treatment. Regarding the sex effect, the aroma and off-odor scores were greater in BARROW than in GILT. Moreover, the off-odor score, as well as the acceptability score, was greater in BARROW vs. GILT only within MPN.

In fresh Boston butt, none of the sensory attributes scored in the present study was influenced by the treatment. The aroma and off-odor scores were greater in GILT than in BARROW whereas the drip score was greater in the latter only within MPN; otherwise, none of the other attributes was influenced by the sex, either. In fresh belly, none of the sensory quality scores was influenced by either the treatment or sex, except for the acceptability score which was greater in BARROW vs. GILT.

In cooked loin, none of the color, aroma, taste, juiciness, tenderness, and acceptability scores differed between MPN and LPN (Table 6). The color score was greater in BARROW than in GILT, but the juiciness, tenderness, and acceptability scores were greater in the latter. The sensory attributes of cooked ham pertaining to meat quality also were not influenced by the treatment. However, scores for all the attributes evaluated in the present study were greater in GILT vs. BARROW, with an exception for the color score which was greater in the former only within LPN.

## Discussion

The LPN, as expected, ate more than MPN during P1. The calculated daily DE and lysine intakes of LPN were 10.5 and 23.5 % lower than those of MPN whereas the ADG and gain:feed ratio of the former were 5.7 and 8.7 % lower than those of the latter. The LPN ate more than MPN during P2 as well; however, the calculated daily DE and lysine intakes of LPN during P2 were 1.4 and 0.2 % greater than those of MPN whereas the ADG and gain:feed ratio of the former were 3.1 and 15.8 % lower than those of the latter. These results suggest that the reduced growth rate and gain:feed ratio of LPN vs. MPN may not have been entirely due to the lower energy or lysine density of the diet provided for the former. In this regard, it is well known that the digestibilities of dietary energy and nitrogen decrease with increasing content of dietary fiber [23]. Further, Chabeauti and Noblet [24] have reported that the energy and nitrogen digestibilities of two fiber-rich diets containing 22 and 44 % wheat bran, respectively, were reduced by 6.6 and 5.1 % and by 14.2 and 12.5 %, respectively, compared with those of a typical corn-soybean meal diet in growing pigs weighing approximately 40 kg. It therefore can be extrapolated from these results that the reduced growth rate and gain:feed ratio of LPN resulted largely from the high wheat bran ratio (30 %) of the diet provided for the group, although the digestibility-reducing effect of the fiber-rich ingredients is known to decrease with increasing age of the animals [23, 25].

The BFT of MPN adjusted for a 115-kg live weight, 24.1 mm, was very close to 24.5 mm of the maximum allowed for the 1^+^ pig carcass grade by the domestic grading standard [18]. Conversely, the predicted live weight of MPN at 22.5 mm of an arbitrary optimum BFT in Korea, which was approximately 108 kg, is thought to be pretty low for modern pork-producing pigs. The experimental animals is thus judged to have been from a genetic lineage with a moderately high BFT as well as a medium weight gain rate as assessed from the BFT results and approximately 750 g of ADG for this line of pigs during the entire grow-finish period on a medium plane of nutrition [26]. As such, the high BFT of the experimental animals was a ‘bottleneck’ in terms of carcass grade and possibly meat quality as well. In this regard, the diet provided for LPN in the present study was effective for increasing the slaughter weight at optimal BFT by virtue of its BFT-lowering effect.

The low plane of nutrition exhibited no advantage over the medium plane of nutrition in any of the physicochemical characteristics measured in the present study. Notably, the a* value of the muscle, which is known to increase with increasing muscular myoglobin content with age [2], did not change in response to the low plane of nutrition although the age of LPN at the time of slaughter was 7 d greater than that of MPN. This was partially different from the results of our previous studies [13, 17] in which this color value increased or did not change in response to the low plane of nutrition in the ham and/or loin. Collectively, it seems plausible that a 7-d difference in slaughter age is marginal to influence the a* value. It was also notable that the decreased shear force measure for the muscle due to the low plane vs. high plane of nutrition observed in the previous study [17] was not detected in the present study. This suggests that differences in shear value may depend on the differentials of the plane of nutrition and possibly growth performance of the groups of animals which are compared.

The LPN exhibited some superiority over MPN in meat quality of fresh ham as indicated by greater color and odor scores in the former, but in other fresh primal cuts, the sensory score was not influenced by the plane of nutrition in any of the attributes evaluated in the present study. It was anticipated in cooked meat that LPN would exhibit greater scores than MPN in flavor-related attributes, because the former was 7 d older than the latter at slaughter and therefore could have greater concentrations of flavoring substances in meat which are known to increase with age [16]. Contrary to this anticipation, neither of cooked loin and ham from LPN exhibited a greater score than that from MPN in any of the sensory attributes. In our previous sensory evaluations on cooked ham and/or loin, LPN did not exhibit any visible advantage over the medium or high plane of nutrition [14, 17], or exhibited only a slightly negative or positive effect [13]. These results suggest that the anticipated beneficial effect of the low plane of nutrition on cooked meat quality is only marginal, or that sensory attributes of cooked muscles without their own associated adipose tissues may not be easily distinguishable by the human palate [16].

The primal cuts from gilts yielded superior physicochemical characteristics, including a lower drip loss and a lower shear force value, than those from barrows. However, the present results need to be confirmed in future studies because the sex effects on physicochemical metrics were rather inconsistent in published studies of ours [12, 13, 17] as well as others [27]. It was also noteworthy that cooked loin and ham from gilts rendered greater scores than those from barrows in the majority of the attributes in sensory evaluation for cooked meat, although in sensory evaluation for fresh meat, the sex effect detected in a few attributes was not consistent among the primal cuts. Results published in the literature regarding the sex effect on sensory attributes of cooked ham and/or loin are varying, ranging from greater scores for gilts vs. barrows in the attributes pertaining to odor and taste [28, 29] to the lack of effect [6, 13, 14, 30] and even greater flavor, juiciness and overall acceptability scores for barrows vs. gilts [27]. These varying results are seemingly reflective of the fact that sensory evaluation is prone to substantial variation by its nature including some confounding between sensory attributes and preferences [31]. Obviously, more studies are needed to make any firm conclusion as to the effect of sex as well as its potential interaction with that of the plane of nutrition on sensory attributes of cooked meat.

## Conclusions

Use of the low plane of nutrition in finishing pigs resulted in decreased BFT and feed conversion ratio compared with growth performance of the animals on the medium plane of nutrition. In addition, the low plane of nutrition exhibited some beneficial effect over that of the medium plane in sensory meat quality of fresh ham, but not in quality of other fresh primal cuts or cooked meat, without influencing physicochemical characteristics of fresh primal cuts. Collectively, the low plane of nutrition may be useful to increase the slaughter weight of finishing pigs with a medium weight gain rate and a moderately high BFT by virtue of its BFT-lowering effect with or without exerting a slightly positive effect on meat quality.

## References

[1] Pettigrew JE, Esnaola MA (2001). Swine nutrition and pork quality: a review. J Anim Sci.

[2] Latorre MA, Lazaro R, Valencia DG, Medel P, Mateos GG (2004). The effects of gender and slaughter weight on the growth performance, carcass traits and meat quality characteristics of heavy pigs. J Anim Sci.

[3] Kim YS, Kim SW, Weaver MA, Lee CY (2005). Increasing the pig market weight: world trends, expected consequences and practical considerations. Asian-Aus J Anim Sci.

[4] Park BC, Lee CY (2011). Feasibility of increasing the slaughter weight of finishing pigs. J Anim Sci Technol.

[5] Lee CY, Kwon OC, Ha DM, Shin HW, Lee JR, Ha YJ (2006). Growth efficiency, carcass quality characteristics and profitability of finishing pigs slaughtered at 130 vs 110 kg. J Anim Sci Technol.

[6] Park MJ, Ha DM, Shin HW, Lee SH, Kim WK, Ha SH (2007). Growth efficiency, carcass quality characteristics and profitability of ‘high’-market weight pigs. J Anim Sci Technol.

[7] NRC (1998). Nutrient Requirements of Swine.

[8] NRC (2012). Nutrient Requirements of Swine.

[9] NSNG (2010). National Swine Nutrition Guide. U.S. Iowa State University.

[10] National Agricultural Statistics Service. http://www.nass.usda.gov/. Accessed 30 Sep 2015.

[11] Ha DM, Park BC, Park MJ, Song YM, Jin SK, Park JH (2012). Effects of plane of nutrition on growth performance and meat quality traits in finishing pigs. J Anim Sci Technol.

[12] Ha DM, Jung DY, Pak MJ, Park BC, Lee CY (2014). Effects of sires with different weight gain potentials and varying planes of nutrition on growth of growing-finishing pigs. J Anim Sci Technol.

[13] Park MJ, Jeong JY, Ha DM, Han JC, Sim TG, Park BC (2009). Effects of dietary energy level and slaughter weight on growth performance and grades and quality traits of the carcass in finishing pigs. J Anim Sci Technol.

[14] Ha DM, Kim GD, Han JC, Jeong JY, Park MJ, Park BC (2010). Effects of dietary energy level on growth efficiency and carcass quality traits of finishing pigs. J Anim Sci Technol.

[15] Lee CY, Lee HP, Jeong JH, Baik KH, Jin SK, Lee JH (2002). Effects of restricted feeding, low-energy diet, and implantation of trenbolone acetate plus estradiol on growth, carcass traits, and circulating concentrations of insulin-like growth factor (IGF)-I and IGF-binding protein-3 in finishing barrows. J Anim Sci.

[16] Lawrence TLJ, Fowler VR, Novakofski JE (1997). Growth of farm animals.

[17] Lee CH, Jung DY, Choi JS, Jin SK, Lee CY (2014). Effects of the plane of nutrition on physicochemical characteristics and sensory quality traits of the muscle in finishing pigs. Korean J Food Sci An..

[18] MAFRA. Grading Standards for Livestock Products (published in Korean). Notification No. 2014–4 of the Ministry of Agriculture, Food and Rural Affairs, Republic of Korea; 2014.

[19] MFAS. Definition of the primal cuts and grades of the carcasses of farm animals (published in Korean). Notification No. 2013–153 of the Ministry of Food and Drug Safety, Republic of Korea; 2013.

[20] On-farm programs. In: Swine Improvement Program Guidelines. National Swine Improvement Federation. http://www.nsif.com/guidel/guidelines.htm. Accessed 3 Aug 2015.

[21] Jin SK, Kim IS, Hur SJ, Hah KH, Kim BW (2004). Effects of feeding period on carcass and objective meat quality in crossbred *longissimus* muscle. J Anim Sci Technol.

[22] CIE. Colorimetry. 2nd ed. CIE Publication No. 15.2, Commision Internationale de l’Eclairage, Vienna; 1986.

[23] Le Goff G, Noblet J (2001). Comparative total tract digestibility of dietary energy and nutrients in growing pigs and adult sows. J Anim Sci.

[24] Chabeauti E, Noblet J, Carre B (1991). Digestion of plant cell walls from four different sources in growing pigs. Anim Feed Sci Technol.

[25] Shi XS, Noblet J (1993). Contribution of the hindgut to digestion of diets in growing pigs and adult sows: effect of diet composition. Livest Prod Sci..

[26] Lee CH, Jung DY, Park MJ, Lee CY (2014). Effects of varying nursery phase-feeding programs on growth performance of pigs during the nursery and subsequent grow-finish phases. J Anim Sci Technol.

[27] Knight CD, Kasser TR, Swenson GH, Hintz RL, Azain MJ, Bates RO (1991). The performance and carcass composition responses of finishing swine to a range of porcine somatotropin doses in a 1-week delivery system. J Anim Sci.

[28] Franco D, Lorenzo JM (2013). Effect of gender (barrow vs. females) on carcass traits and meat quality of Celta pig reared outdoors. J Sci Food Agri.

[29] Klindt J, Buonomo FC, Yen JT (1995). Administration of porcine somatotropin by sustained-release implant: growth, carcass, and sensory responses in crossbred white and genetically lean and obese boars and gilts. J Anim Sci.

[30] Lammers PJ, Kerr BJ, Weber TE, Bregendahl K, Lonergan SM, Prusa KJ (2008). Growth performance, carcass characteristics, meat quality, and tissue histology of growing pigs fed crude glycerin-supplemented diets. J Anim Sci.

[31] Risvik E (1994). Sensory properties and preferences. Meat Sci.

